# Insights into the Microstructure of Hydrothermal Synthesized Nanoscale K_2_O-Al_2_O_3_-SiO_2_-H_2_O Particles

**DOI:** 10.3390/nano10010063

**Published:** 2019-12-26

**Authors:** Bao Liu, Chunyan Zhu, Kunde Zhuang, Le Shuai, Dongxu Li, Wujian Long, Feng Xing, Yuan Fang

**Affiliations:** 1 College of Civil and Transportation Engineering, Shenzhen University, Shenzhen 518060, China; baoliu@njtech.edu.cn (B.L.); zcy243003@163.com (C.Z.); vensen2261109@foxmail.com (K.Z.); 1810332025@email.szu.edu.cn (L.S.); dongxuli@njtech.edu.cn (D.L.); longwj@szu.edu.cn (W.L.); xingf@szu.edu.cn (F.X.); 2 Guangdong Provincial Key Laboratory of Durability for Marine Civil Engineering, Shenzhen 518060, China; 3 Jiangsu National Synergetic Innovation Center for Advanced Materials (SICAM), Nanjing Tech University, Nanjing 210009, China

**Keywords:** K-A-S-H, zeolite, nuclear magnetic resonance, polymerization degree, phase composition

## Abstract

K-A-S-H (K_2_O-Al_2_O_3_-SiO_2_-H_2_O) gel is a key phase that forms in most alkali-activated binders (eco-friendly binders which utilize a substantial amount of industrial by-product). An in-depth understanding of the microstructure and performance of this nano-sized key phase facilitates better application to alkali-activated binders. However, such studies remain little and undetailed. Therefore, our research aims to provide insights into the microstructure of K-A-S-H particles synthesized with accurate stoichiometric control by the hydrothermal method through thermogravimetric analysis (TG), Fourier transform infrared spectroscopy (FTIR), nuclear magnetic resonance (NMR), scanning electron microscopy (SEM), transmission electron microscopy (TEM) and BET surface area. The results show that for materials prepared at the curing temperature lower than 80 °C, the K-A-S-H products were completely amorphous. With increased curing temperature and time, the K-A-S-H products were transformed from the amorphous phase to the crystalline zeolite phase structure, with a reduction in the specific surface area. The TG results indicate that the crystalline phase contains more non-evaporated water or zeolite water for structural rearrangement. The degree of tetrahedral polymerization slightly decreased with an increase of the K_2_O/SiO_2_ ratio as the amount of non-bridged oxygen atoms increased, whereas it gradually increased with an increase of curing temperature and time, as suggested by the FTIR and NMR results. Various K_2_O/SiO_2_ ratios resulted in the formation of zeolite K-H and K-G zeolite, both of which exhibited highly polymerized three-dimensional network structures. However, there was no significant effect of the SiO_2_/Al_2_O_3_ ratio on the structure of K-A-S-H products. Overall, these results provide insight into understanding the chemical stability of K-A-S-H.

## 1. Introduction

The increasing demand for low-cost and durable construction materials has stimulated the study of alternative cementitious binders [1]. Alkali-activated binders have been widely discussed and promoted. These binders are promising cementitious materials that can be used in place of Portland cement (PC) in many applications, including concrete. Compared with Portland cement-based production, the use of alkali-activated materials can reduce related carbon dioxide emissions by more than 80% [2,3], an important concern worldwide. At the same time, this research is important for the reuse of solid waste. The properties of alkali-activated binders are dependent on the type of gel that dominates the structure, which is largely determined by the calcium content in the system. A primary reaction product of alkali-activated binder systems may be an alkali aluminosilicate–type gel [4], a gel type that is poor in calcium. This gel is often represented as hydrated sodium aluminosilicate N-A-S-H (N_2_O-Al_2_O_3_-SiO_2_-H_2_O) or, with substitution of potassium for sodium, K-A-S-H (K_2_O-Al_2_O_3_-SiO_2_-H_2_O) [5]. This nomenclature describes the chemical nature of the reaction product rather than its precursor, similar to the designation of C-S-H (CaO-SiO_2_-H_2_O) [6].

Despite the promising qualities of these materials, the use of alkali-activated materials in the construction industry remains limited, partly because of the complex and volatile chemical composition of wastes. Additionally, because by-product materials are used as the main precursors, it is difficult to achieve reproducibility and accurate stoichiometry of the main hydration products formed in these systems. To solve these problems, a better understanding of the reaction mechanism of the alkali-activated binder system is required. For this reason, many recent studies have explored the artificial synthesis of gels with pre-designed compositions. Our previous research [3,4,7] performed the hydrothermal synthesis of C-A-S-H (CaO-Al_2_O_3_-SiO_2_-H_2_O) and N-A-S-H and showed that with a controlled composition of raw materials, the resulting product has high purity and is similar in composition with alkali-activated product gel. The temperature and curing time are important factors determining the characteristics of the prepared materials, resulting in different microstructure and performance of alkali-activated cementitious material hydration products. Walkley et al. [2] also demonstrated the production of stoichiometrically controlled N-A-S-H gels via alkali-activation of high-purity synthetic amorphous aluminosilicate powders, providing an innovative process to accurately simulate the chemistry of alkali-activated cementitious materials. Constructed models based on those for zeolite, primarily related to analcime, were also proposed, in which assumes that the (N, K)-A-S-H gel is amorphous and has a randomly distributed cluster reciprocal structure with short-range order of SiO_4_ tetrahedron and AlO_4_ octahedron [8,9]. However, it was argued that in the N-A-S-H structure, both Si and Al exist in the form of coordinated tetrahedrons, whereas Si is mainly in the Q_4_ (mAl) environment with the m value depending on the Si/Al in the structural composition [10,11]. Additionally, the N-A-S-H chemical composition is similar to the hydrothermal synthesis of zeolite, with a structure that is characterized by short-range order. Sol-gel synthesis method was also adopted [12] and a model was established in which colloids can form crosslinks to fabricate a three-dimensional network structure. The use of an Al_2_O_3_/K_2_O ratio of 1.0 is required because certain other ions must be used to offset the electrical imbalance in the structure when Al^3+^ ions replace the Si^4+^ ions in the polymer. These studies [10,11,13,14] on the structural composition and synthesis of N-A-S-H gel revealed a three-dimensional network structure formed by a common oxygen atom between an aluminoxy tetrahedron and a siloxane tetrahedral structural unit. The cavity formed inside the three-dimensional network structure can be filled with the equilibrium structural charge of the alkali metal cations, Na^+^ or K^+^.

However, the location of alkali metal cations in gels or the retention mechanisms involved were not completely resolved in the above-mentioned studies. Although both K-A-S-H and N-A-S-H are hydration products of low-calcium alkali-activated materials, the differences in the solvated ionic radius and enthalpy for the hydration of K and Na cause these materials to exhibit different affinities for negatively charged surfaces [15,16,17]. These different affinities cause a large difference in the chemical composition and structure of the resulting gel. Little is known about the synthesis and performance characterization of K-A-S-H-based nanogels. Thus, the goal of this work was to evaluate the chemical composition and structure of K-A-S-H gels and explore the state of Al in the inner structure of these gels.

In this study, the products of potassium aluminosilicate formed under hydrothermal conditions include aluminosilicate gel or zeolite. The products were characterized by Fourier transform infrared spectroscopy (FTIR), thermogravimetric analysis (TG), scanning electron microscopy (SEM), and transmission electron microscopy (TEM). Unfortunately, due to the poor crystallization of most gel phases, X-ray diffraction (XRD) cannot provide structural information. As an alternative, we adopted advanced characterization techniques, including ^27^Al, ^29^Si magic angle spinning-nuclear magnetic resonance (MAS-NMR) analysis of atomic coordination and polyhedral polymerization in crystalline and amorphous materials. The results from this work approximately reflect the changes in the composition and structure of hydration products in the alkali-activated cementitious materials due to temperature, time, and the composition of the raw materials. These findings can approximately reflect the changes in the composition and structure of the alkali-activated binders under various synthetic conditions and can provide a better understanding of the phase transformations and microstructure development of alkali-activated cementitious materials.

## 2. Experimental Procedure

### 2.1. Materials

Nano-SiO_2_ (50 ± 5 nm) and nano-Al_2_O_3_ (γ-phase, 10 nm) were purchased from Macklin Biochemical Co., Ltd. (Shanghai, China). Potassium hydroxide (KOH) and anhydrous ethanol were obtained from Xilong Scientific Co., Ltd. (Guangdong, China). Deionized water was used to synthesize K-A-S-H. All materials were of analytical reagent (A.R.) grade.

### 2.2. Synthesis of K-A-S-H

The proportions of nano-SiO_2_, nano-Al_2_O_3_, KOH, and deionized water are shown in Table 1. Nanoscale K-A-S-H gel was synthesized according to our previous hydrothermal method [3,4]. The samples are named as follows: using K-A-S-H1.0_1_-1d-95 as an example, the K-A-S-H1.0 indicates a K_2_O/SiO_2_ ratio in the K-A-S-H system of 1.0, the subscript indicates a SiO_2_/Al_2_O_3_ ratio of 1, the 1d indicates a curing time of 1 day, and the 95 indicates that the material was cured at a temperature of 95 °C.

### 2.3. Characterization of Samples

The XRD patterns of K-A-S-H powder were obtained on a Brucker D8 Advance X-ray diffractometer (Brucker, Karlsruhe, Germany). Thermogravimetric analysis of K-A-S-H powder was performed using a Perkin Elmer Diamond instrument (Boston, MA, USA). The polymerization state analyses utilized the techniques of Fourier transform infrared (FTIR, PerkinElmer, Boston, MA, USA) and ^29^Si, ^27^Al solid-state MAS-NMR. The morphology analyses were performed using scanning electron microscopy (SEM, FEI Company, Hillsboro, OR, USA) and transmission electron microscopy (TEM, JEOL, Tokyo, Japan). A DelsaMax CORE nanolaser particle size analyzer (Beckman Coulter, Shanghai, China) was used to determine the particle size distribution of K-A-S-H samples.

## 3. Results and Discussion

### 3.1. X-Ray Diffraction Analysis

**Figure 1:**
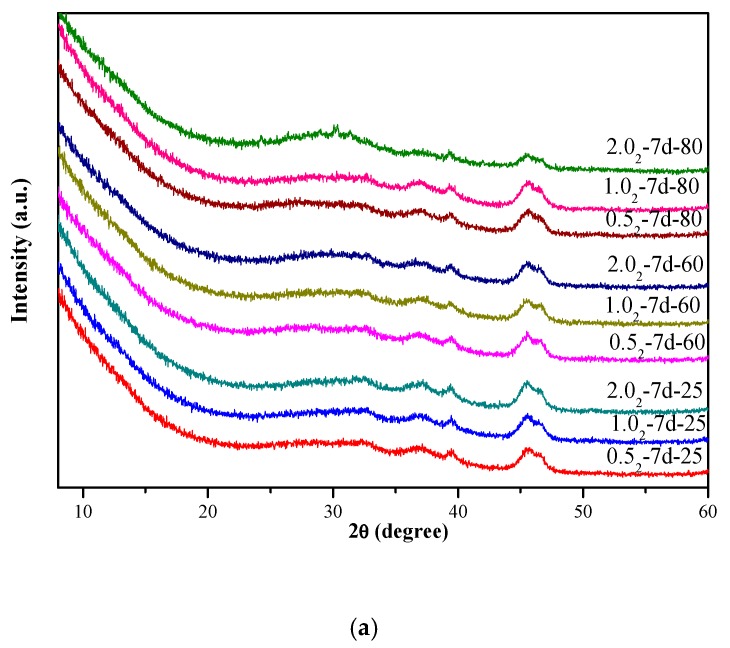

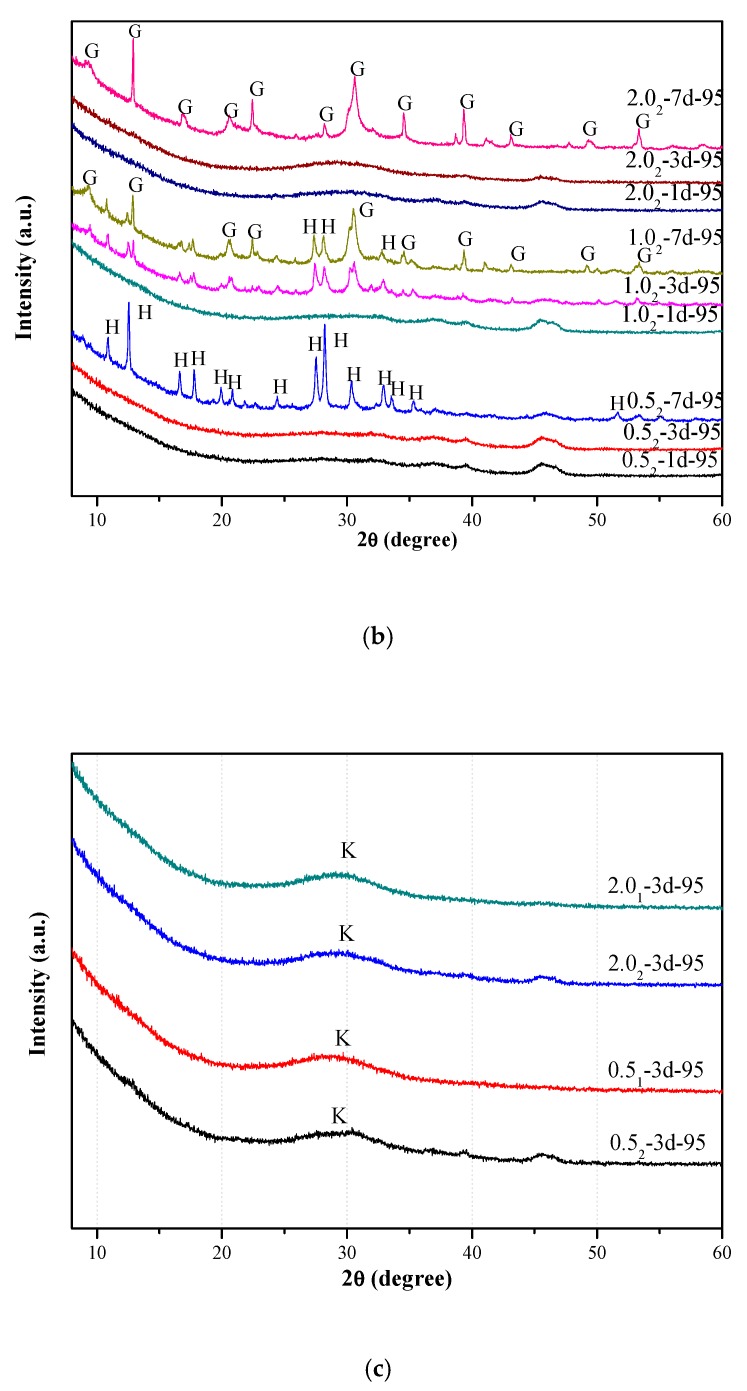
X-ray diffraction (XRD) patterns of K-A-S-H samples (**a**) synthesized at different reaction temperatures, (**b**) synthesized by reaction at 95 °C, and (**c**) synthesized using different SiO_2_/Al_2_O_3_ ratios at 95 °C. (K: K-A-S-H gels, G: K-G zeolite, H: Zeolite K-H).

Figure 1 shows the XRD patterns of the K-A-S-H samples. At 25 °C, 60 °C, and 80 °C curing temperature, all the prepared K-A-S-H products appeared completely amorphous. The K_2_O/SiO_2_ ratio and curing time had no significant effect on the crystallinity of the samples, as shown in Figure 1a. In addition, a broad featureless hump caused by amorphous aluminosilicate was observed between 25° and 35° 2θ [4]. As the curing temperature increased to 95 °C, all K-A-S-H products remained amorphous at the 1-day age. When the curing time reached 3 days for K-A-S-H1.0_2_-3d-95, crystalline phases first appeared, though the crystallinity of the K-A-S-H samples prepared using other K_2_O/SiO_2_ ratios was still very poor. When the curing time was extended to 7 days, all K-A-S-H samples were transformed into the crystalline state, as shown in Figure 1b. Specifically, The XRD pattern (see Figure 1b) of the crystal phase formed by K-A-S-H0.5_2_-7d-95 closely matched that of zeolite K-H, with PDF#16-0692, K_2_Al_2_Si_4_O_12_•*x*H_2_O, Si:Al:K = 2:1:1, which is classified as an unknown structure type. Similar results were also reported by Sathupunya et al. [18] and Liu et al. [19]. In addition, K-G zeolite (K_2_Al_2_SiO_6_•H_2_O, PDF#12-1094) [20] crystalline phase was observed in the sample K-A-S-H2.0_2_-7d-95, and weak diffraction peaks of zeolite K-H and K-G zeolite were observed in sample K-A-S-H1.0_2_-3d-95. With an increase of curing time, the degree of crystallinity increased. This may be attributed to the difference in the crystalline structure and category of the zeolite phase induced by the difference in K_2_O content. Yuan et al. [20] found that zeolite K-H is a stable phase at lower concentrations of KOH, and K-G zeolite is more stable at relatively higher concentrations of KOH. In summary, K^+^ and OH^-^ are the dominant influence factors controlling the types of zeolite formed in the products. However, as shown in Figure 1c, SiO_2_/Al_2_O_3_ ratios had no significant effect on the crystallinity of the products. Moreover, the product X-ray diffraction pattern at 95 °C for material aged for 3 days showed a hump at 2θ of 25–35°, similar to the amorphous hump and low crystalline phase seen for (N, K)-A-S-H gels [21,22].

### 3.2. Thermogravimetric Analysis

**Figure 2:**
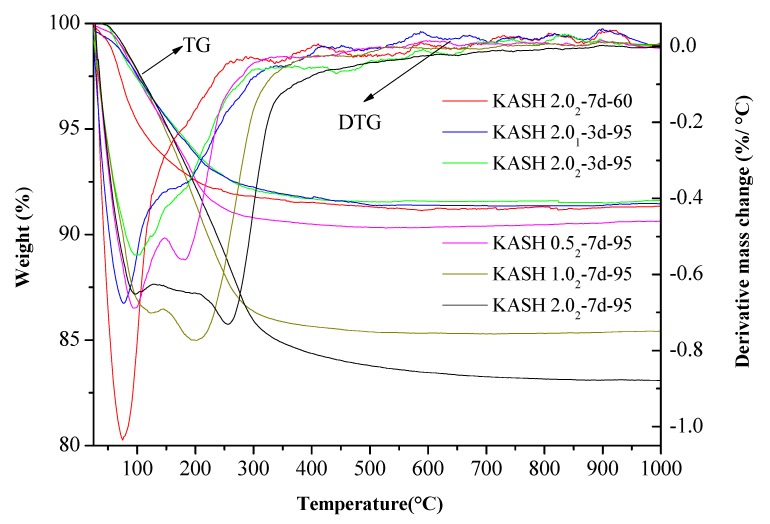
TG/DTG curves of K-A-S-H samples.

The TG/DTG curves of the K-A-S-H samples are shown in Figure 2. In general, each K-A-S-H powdery sample showed a mass loss below 150 °C. This mass loss value was related to changes in the amount of water adsorbed or evaporated [23] during synthesis. There was a large mass loss for sample K-A-S-H2.0_2_-7d-60, suggesting high adsorbed water content due to incomplete reaction of the raw materials of the sample under the curing condition of 60 °C (the raw materials were highly hygroscopic [2]). Samples synthesized using three different K_2_O/SiO_2_ ratios were cured for 7 days at 95 °C, and the TG/DTG curves showed small mass loss peaks at 190 °C, 200 °C, and 250 °C, separately. Combined with the XRD results, the samples contained crystalline zeolite phases, indicating more tightly physically bound and/or loss of zeolite water [2,24]. These peaks were not present before 7 days of curing time, which indicates that rearrangement and structural ordering of the water-containing phases (e.g., zeolites) persisted from the 3rd day to the 7th day of curing time as the reaction progressed. Samples K-A-S-H2.0_1_-3d-95 and K-A-S-H2.0_2_-3d-95 also showed small mass loss peaks at around 200 °C in the TG/DTG curves, due to the loss of nonevaporable water [23,24], as defined by Thomas et al. [25,26]. This can be associated primarily with structural water and some constrained water in the gel.

As most of the sample was amorphous K-A-S-H, the aforementioned techniques were unable to fully reveal its structural information, especially its main structural unit [SiO4]^4−^ tetrahedron. It is crucial to understand the polymerization process of [SiO4]^4−^ tetrahedron, as it will provide an understanding of the formation mechanism of the nanostructures of these gels [20,27,28]. Therefore, in the next sections, infrared spectroscopy (FTIR) and nuclear magnetic resonance (NMR) were used to study the [SiO4]^4−^ tetrahedral anion polymerization state and aluminum coordination state in the K-A-S-H products.

### 3.3. Infrared Analysis

**Figure 3:**
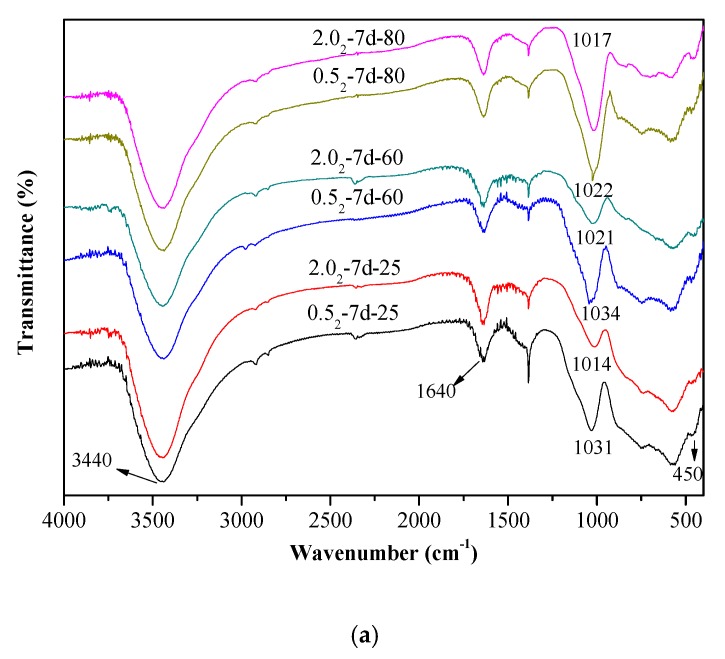

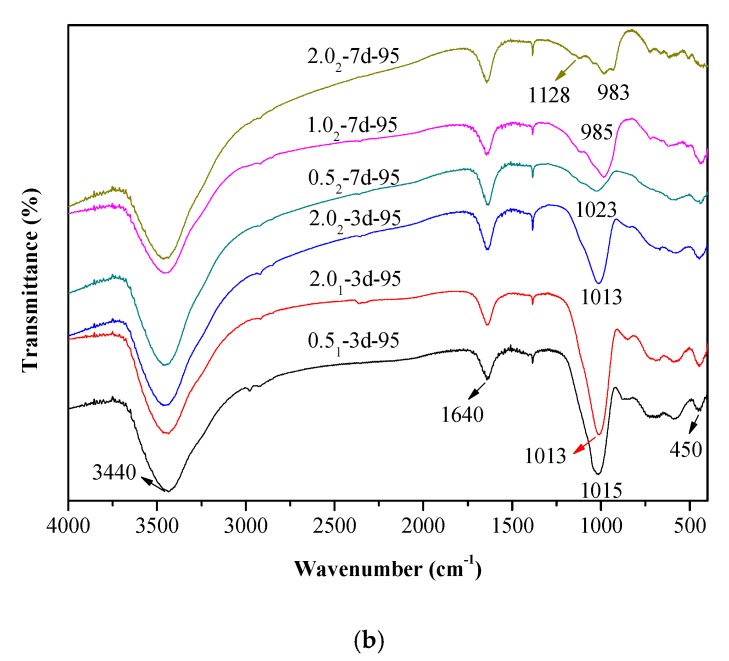
Fourier transform infrared spectroscopy (FTIR) spectra of K-A-S-H samples (**a**) synthesized by reaction at temperatures lower than 95 °C, (**b**) synthesized by reaction at 95 °C.

The vibrational spectra of different K-A-S-H samples were determined and are shown in Figure 3. The characteristic bands at 3440 cm^−1^ and 1640 cm^−1^ are respectively caused by the stretching vibration and bending vibration of O–H in adsorbed water [29,30]. The formation of Si–O–T bonds (T = tetrahedral Si or Al) results in a strong stretching vibration signal at 1031 cm^−1^ [2], but this bond is located at about 950 cm^−1^ in the chain structure of C-(A)-S-H [31]. The K-A-S-H gels show quite different results. The main band (νas T–O: where T is Si or Al) moves from a low to high wavenumber position, which is more typical in a low calcium environment gel. The characteristic peak of the stretching vibrations of T–O–Si (T Si or Al) bonds is located at about 1031 cm^−1^ in the K-A-S-H sample at 25 °C, as shown in Figure 3a. With increased curing temperature, this band only slightly shifts to 1021 cm^−1^. When the curing temperature was increased to 95 °C (see Figure 3b), this characteristic peak showed a significant shift to 1013 cm^−1^. This shift was also seen with increased curing time, and this band in sample K-A-S-H2.0_2_-7d-95 shifted to 983 cm^−1^. The shifts occur with increased silicon substitution by aluminum in the second coordination sphere due to the weaker Al–O bonds [14]. In addition, the main bands, corresponding to the T–O–Si (T Si or Al) stretching vibrations in the K-A-S-H samples, became somewhat rounder and broader in shape, particularly after curing at 95 °C for 7 days. The change of polarization of the oxygen atom on the Si–O–Al linkage, may cause an increase in the remaining Si–O bond strength in the previous pairing silicate tetrahedron. This may lead to a higher wavenumber contribution to the main Si–O band, thus broadening the band around 983 cm^−1^ [32]. Comparison of the position of the T–O–Si stretching vibration characteristic peak for different K_2_O/SiO_2_ ratios reveals band shifts. For materials at the same SiO_2_/Al_2_O_3_ ratio, as the K_2_O/SiO_2_ ratio increased, the band shifted to a low wavenumber. This indicated that an increase in K_2_O/SiO_2_ ratio causes a decrease in TO_4_ (T is Si or Al) tetrahedral polymerization. These observations are similar to the formation [2,23,28] of the N-A-S-H system product linkages. When K_2_O/SiO_2_ is increased, the amount of K^+^ in the reaction system exceeded the quantity required to balance the negative charges of AlO^4−^ tetrahedra. To maintain electric neutrality, the number of non-bridging oxygen bonds with a negative charge in the structure increases. The non-bridging oxygen bonds are not shared by two or more polyhedrons, only connected with a net-forming ion, and the original structure of TO_4_ (T is Si or Al) becomes more isolated [2,33,34]. As a result, the band of Si–O–T stretching vibration moves to a lower wavenumber in the FT-IR spectrum. With increased curing time or temperature, the band appears at a lower wavenumber, indicating that excess K^+^ in the reaction system entered the aluminosilicate structure with an increased reaction degree.

Additionally, a characteristic peak at 1128 cm^−1^ was present in the samples cured at 95 °C for 7 days. This vibrational band at 1128 cm^−1^ decreased with decreased K_2_O/SiO_2_ ratio due to the transformation of octahedral Al to tetrahedral Al and the formation of Si–O–Al as Al combined with Si–O–Si [20]. Compared with the T–O–Si (T = Si or Al) stretching vibrations in other K-A-S-H samples, the main bands became somewhat rounder in shape and appeared broader. At constant K_2_O/SiO_2_ ratio, a change in the SiO_2_/Al_2_O_3_ ratio did not significantly change the wavenumber of the characteristic stretching peak of T–O–Si (T = Si or Al) asymmetric stretching vibration (see Figure 3b), this was consistent with the XRD results and indicated little significant effect of the SiO_2_/Al_2_O_3_ ratio for T–O–Si (T = Si or Al) stretching vibration frequency. The bands with wavenumbers ranging from 800 to 600 cm^−1^ are mainly caused by Al–O stretching vibrations [33,34], and no significant difference was observed in the position of these bands. The absorption peak at 450 cm^−1^ is mainly attributed to the bending vibration of Si–O–Si and Si–O–Al, and this weak peak is present in all samples at approximately the same position. We will discuss this below in the analysis of the ^29^Si and ^27^Al MAS-NMR results.

### 3.4. ^29^Si and ^27^Al MAS-NMR Analysis

Determination of the coordination number of aluminums with oxygen (four, five, or six) is possible using ^27^Al MAS-NMR and ^29^Si MAS-NMR methods to differentiate between the different SiQ^n^(mAl) structural units [14,35].

**Figure 4:**
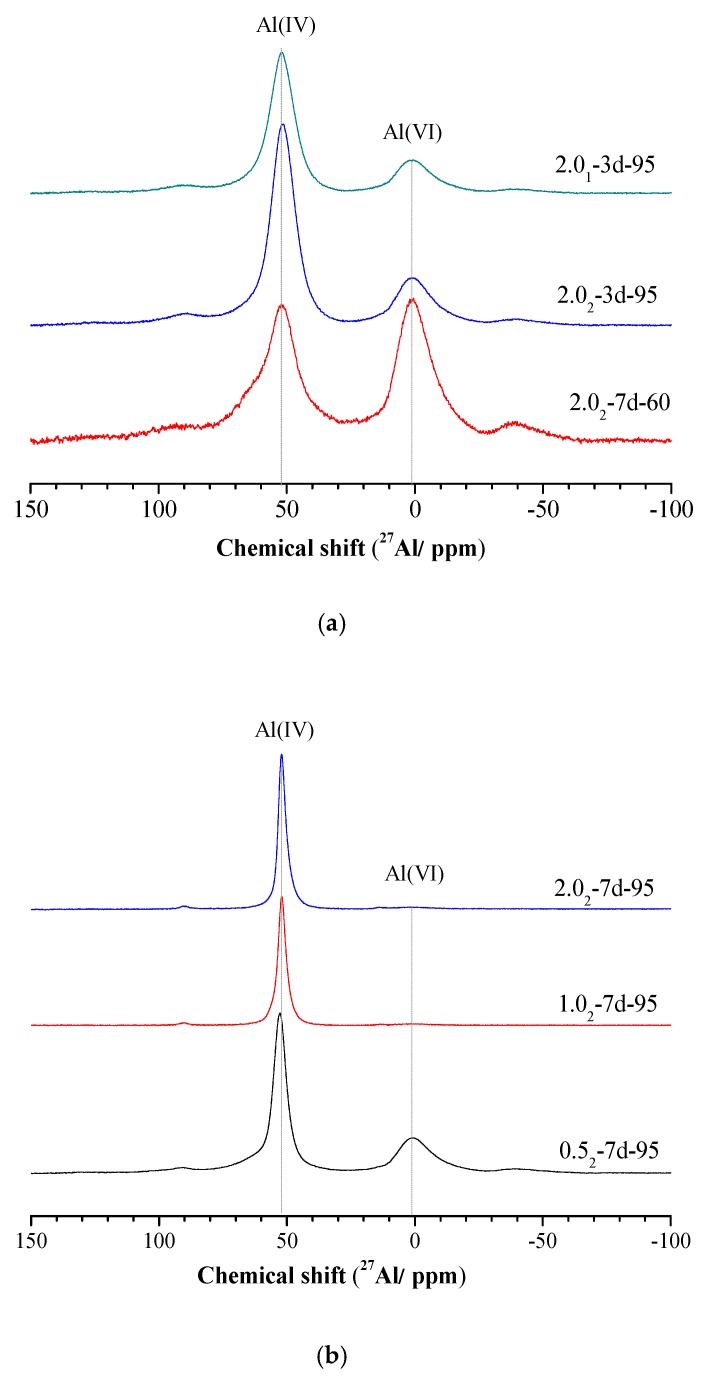
^27^Al MAS-NMR spectra of K-A-S-H samples (**a**) synthesized at different reaction temperatures and SiO_2_/Al_2_O_3_, (**b**) synthesized using different K_2_O/SiO_2_ ratios by reaction at 95 °C.

The ^27^Al MAS-NMR spectra of different samples were determined and are shown in Figure 4. For amorphous or semi-crystalline K-A-S-H products, obtained ^27^Al MAS-NMR spectra can be split into two asymmetric lines with line shapes that are typical of Al in an amorphous environment (Figure 4a,b). The chemical shifts at 52 ppm and 1 ppm are respectively related to Al in tetrahedral (noted as Al _IV_) and hexahedral environments [36,37].

The variation of the SiO_2_/Al_2_O_3_ ratio did not significantly change the structure of the obtained K-A-S-H samples. However, increasing the curing temperature changed the process of aluminum coordination, from VI coordination to IV coordination, as shown in Figure 4a. By extending the age of curing, the weak peak at 1 ppm slowly weakened until it disappeared, and the spectra for all the K-A-S-H samples after 3 days were much wider than those for the products cured for 7 days (see Figure 4). Additionally, it can be seen that the reduction of the SiO_2_/Al_2_O_3_ ratio favors the proportion of Al _VI_ to the detriment of Al _IV_. A lower K_2_O/SiO_2_ ratio may increases the percentage of Al _VI_ involved in the sialate (–Si–O–Al–O–) tetrahedral crosslink [2]. Davidovits et al. [6] assume that K^+^ compensates for a weak negative charge on Al _IV_, and this is linked with a strong ionic bond within the Si–O–Al network. In summary, curing temperature and time showed a great effect on the morphology of ^27^Al MAS-NMR spectra. The NMR spectroscopy shows an increased degree of polymerization with increased curing temperature and time for all K-A-S-H samples [36].

**Figure 5:**
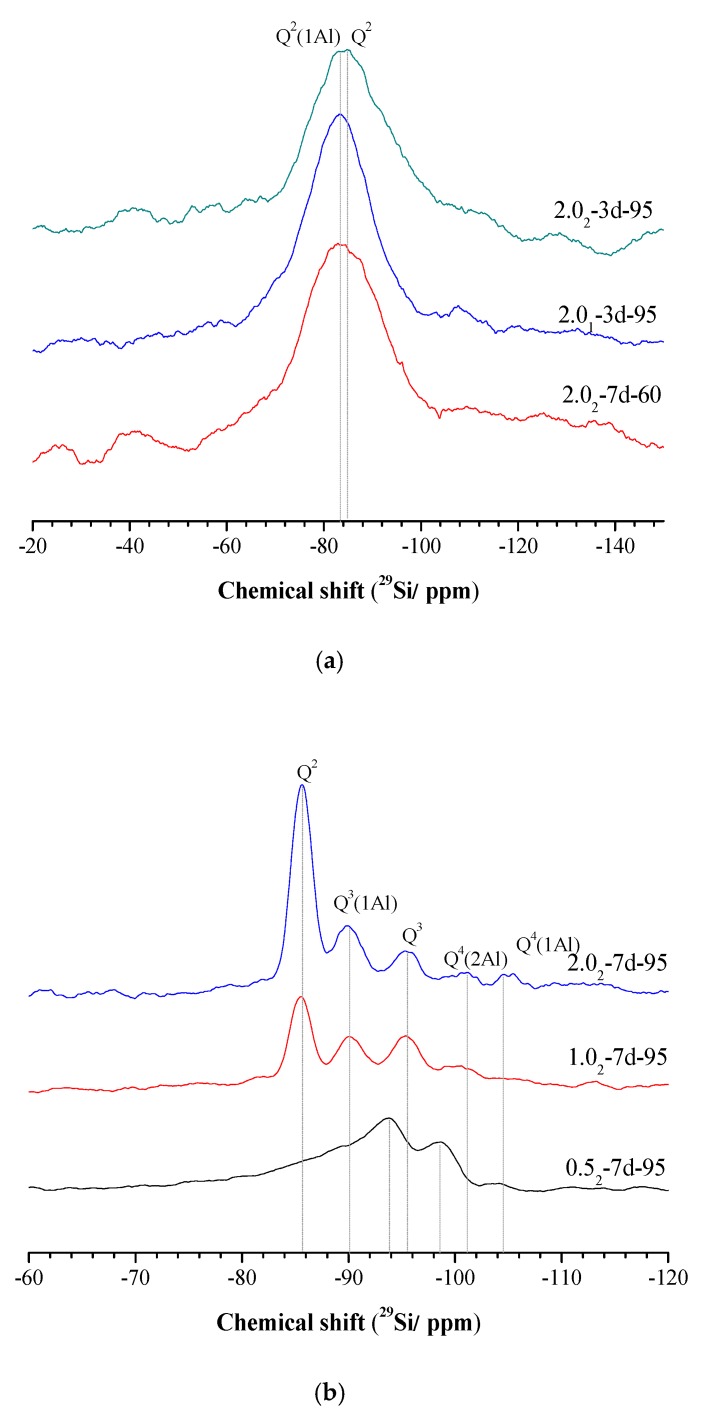
^29^Si MAS NMR spectra of K-A-S-H samples (**a**) synthesized at different reaction temperatures and different SiO_2_/Al_2_O_3_ ratios, and (**b**) synthesized using different K_2_O/SiO_2_ ratios by reaction at 95 °C.

The ^29^Si MAS-NMR spectra of different samples are shown in Figure 5. The change of SiO_2_/Al_2_O_3_ ratio (with K_2_O/SiO_2_ ratio kept constant) has little effect on the morphology of the ^29^Si MAS-NMR spectra of the samples. When the curing temperature was increased by 95 °C, Q_2_ shifted to the negative direction, as shown in Figure 5a. As shown, there is only one main peak, with the first resonance at −83 ppm and the second at −86 ppm. These peaks have been attributed to Q_2_ (1Al) and Q_2_ (0Al) units, respectively, indicating that most silicate tetrahedra are chain mid-members and Al substitution for Si has occurred in the silicate chains [36,37].

After curing at 95 °C for 7 days, the ^29^Si MAS-NMR chemical shift moved to a negative value, as shown in Figure 5b. This suggests a certain degree of structural rearrangement and greater cross-linking [2] in the K-A-S-H samples due to the formation of Si-O-Si bonds, and increased degree of polymerization with increased curing time and temperature. Under this condition, five characteristic peaks appear at a high K_2_O/SiO_2_ ratio, with chemical shifts that correspond to −85.5 ppm, −90 ppm, −95.5 ppm, −101 ppm, and −104.5 ppm. This is indicative of the formation of a highly polymerized, highly Al-substituted Si environment. A similar phenomenon was also observed in (C, N)-A-S-H gels [2,24]. The −85 ppm shift corresponds to Q_2_ units. The chemical shifts at −90 ppm and −95 ppm correspond to Q_3_, and are consistent with chain branching sites (Q_3_ (1Al) units) and three-dimensional cross-linked sites (Q_3_ units), both with substantial Al substitution for Si [14]. The chemical shifts of −101 ppm and −104.5 ppm were assigned to Q_4_ (2Al) and Q_4_ (1Al), respectively. The samples prepared with a high K_2_O/SiO_2_ ratio are thus characterized as a highly polymerized structure composed principally of three-dimensionally cross-linked sites such as Q_4_ (2Al) and Q_4_ (1Al) units. As the K_2_O/SiO_2_ ratio decreased, the two peaks slowly weakened in intensity. When the K_2_O/SiO_2_ ratio decreased to 0.5, the ^29^Si MAS-NMR chemical shifted to a more negative value. The chemical shifts of −94 ppm and −99 ppm was respectively assigned to Q_3_ and Q_4_ (3Al), and a weak peak at 104.5 ppm was attributed to Q_4_ (1Al) [14,38] (see Figure 5b). This indicated that at different K_2_O/SiO_2_ ratios, K-A-S-H samples have different highly polymerized three-dimensional network zeolite structures after treatment at a certain curing temperature and time, with the higher K_2_O/SiO_2_ ratio leading to a more developed framework. This finding is consistent with the XRD and ^27^Al MAS NMR analysis of these samples, and is further confirmed by visual observation in the SEM images described below.

In summary, amorphous or semi-crystalline K-A-S-H samples were formed from longer chains but the ^29^Si MAS-NMR spectra showed mainly chain mid-member sites [Q_2_ and Q_2_ (1Al)] [39]. In contrast, crystalline K-A-S-H samples mainly contain three-dimensional cross-linked sites [Q_4_ (2Al) and Q_4_ (1Al)], reflecting a highly polymerized structural aluminosilicate framework [40]. The above results are in agreement with the conclusions from the infrared and ^27^Al MAS-NMR spectroscopy results that increasing the curing temperature and time result in the continuous increase of the degree of polymerization of samples [36].

### 3.5. SEM Analysis

**Figure 6:**
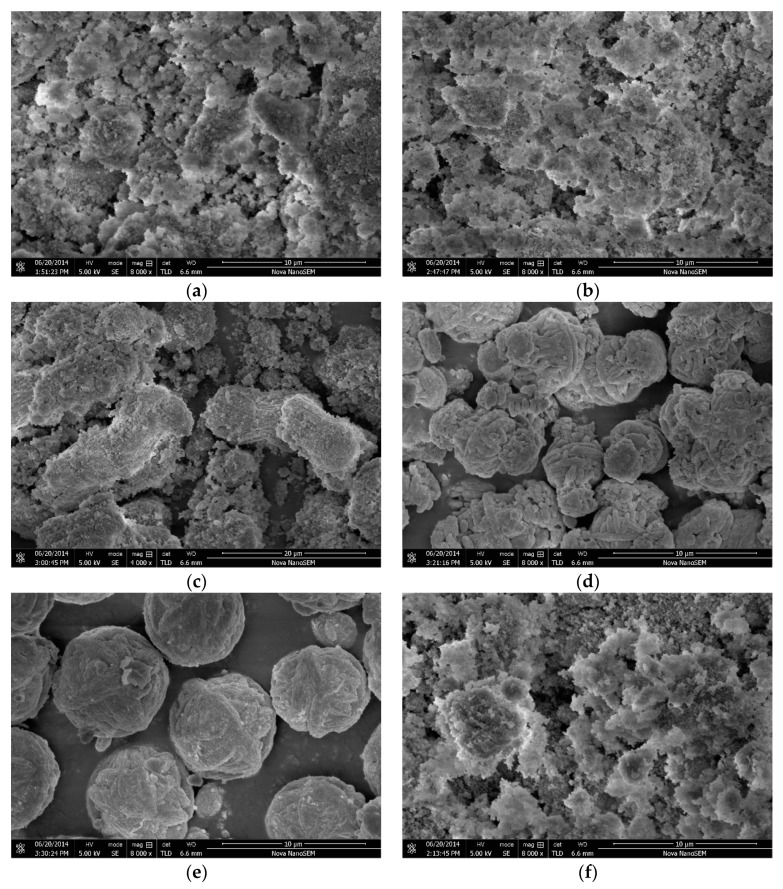
SEM images of K-A-S-H samples (**a**) K-A-S-H2.0_2_-3d-60, (**b**) K-A-S-H2.0_2_-3d-95, (**c**) K-A-S-H0.5_2_-7d-95, (**d**) K-A-S-H1.0_2_-7d-95, (**e**) K-A-S-H2.0_2_-7d-95, and (**f**) K-A-S-H2.0_1_-3d-95.

Figure 6 shows the scanning electron micrographs of the K-A-S-H samples. For curing temperature lower than 80 °C, it can be clearly seen that the reaction products are mainly amorphous K-A-S-H products. These products exhibit a loose flocculent form and are covered with fine powder (see Figure 6a), which is likely unreacted raw materials. When the curing temperature was increased to 95 °C, within 7 days of curing, most products remained in the irregularly flocculent form, with relatively loose filling, as shown in Figure 6b. However, we were unable to fully distinguish an amorphous gel. Thus, in Section 3.6, TEM image analysis was applied. At a reduced SiO_2_/Al_2_O_3_ ratio, a small amount of crystal phase particles appeared wrapped by amorphous gel particles or filled between particle gaps (see Figure 6f). In addition, for the products cured at 95 °C for 7 days, there were more obvious crystallized particles. At a K_2_O/SiO_2_ ratio of 0.5, the number of crystal particles increased, there were many thin strips of crystals that overlapped each other to form cluster-like crystals, and flocculent small particles were apparent on the surface (see Figure 6c). The Si:Al:K ratio of the product was 1.88:1:0.95, as determined via EDS, consistent with the formula for zeolite K-H synthesized by the sol-gel method and the previous measurement of 1.98:1:0.82 [18,19]. The image of the sample prepared with a K_2_O/SiO_2_ ratio of 2.0 and cured at 95 °C is shown in Figure 6e. The crystal particles appeared fuller and denser, there were spherical crystals formed by the cross-linking of fibers and strips, and the crystallized particles were about 6–8 μm in diameter. EDS analysis indicated a Si:Al:K ratio of the product that was close to the atomic ratio of K-G zeolite [20]. However, at a K_2_O/SiO_2_ ratio of 1.0, the crystal particles are interspersed and chimeric with many cluster-like crystals and spherical crystals, consistent with the XRD results that there are two crystal phases of zeolite K-H and K-G zeolite. Besides, the change of micromorphology observed in a zeolite (Figure 6c–e) may be attributed to the different polarizability of K^+^ ions [41].

### 3.6. TEM Analysis

**Figure 7:**
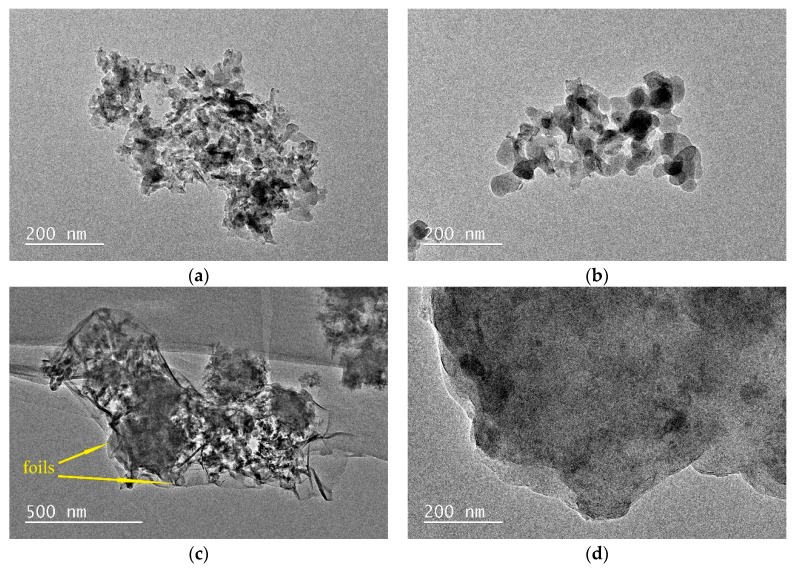
TEM images of K-A-S-H samples (**a**) K-A-S-H 2.0_2_-7d-60, (**b**) K-A-S-H 2.0_2_-3d-95, (**c**) K-A-S-H 0.5_2_-7d-95, (**d**) K-A-S-H 2.0_2_-7d-95.

Figure 7a shows the TEM image of sample K-A-S-H2.0_2_-7d-60, revealing mainly a loose flaky structure with aggregated strip particles and a porous and rough surface, surrounded by some spherical particles, which should be amorphous gels. Similar results were also reported by Pena et al. [42]. When the curing temperature was increased to 95 °C for sample K-A-S-H 2.0_2_-3d-95, as shown in Figure 7b, the products showed mainly irregular spherical particles of about 50–100 nm. The particles overlapped each other to form a small piece, different from sample K-A-S-H2.0_2_-7d-60. Considered with the results of XRD analysis, in most cases, a significant amount of amorphous K-A-S-H gels was detected. For the sample cured for 7 days, K-A-S-H 0.5_2_-7d-95 (see Figure 7c), there were no crystal grains, and thin, crumpled foils were observed [24], with some fibrous structures of about 200 nm polymerized together with the foils. Additionally, a small amount of flaky structure indicated the presence of the crystalline phase in the samples. For the sample prepared at a K_2_O/SiO_2_ ratio of 2.0 at this curing temperature, as shown in Figure 7d, the layered sheet structure indicates the presence of another crystalline phase in the samples that is denser than that in the samples prepared at the low K_2_O/SiO_2_ ratio.

### 3.7. Particle Size Analysis

**Figure 8:**
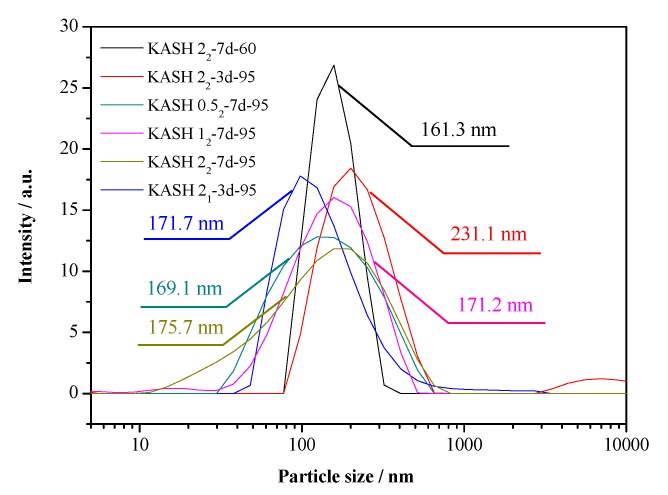
Particle size distribution of K-A-S-H samples.

**Figure 9:**
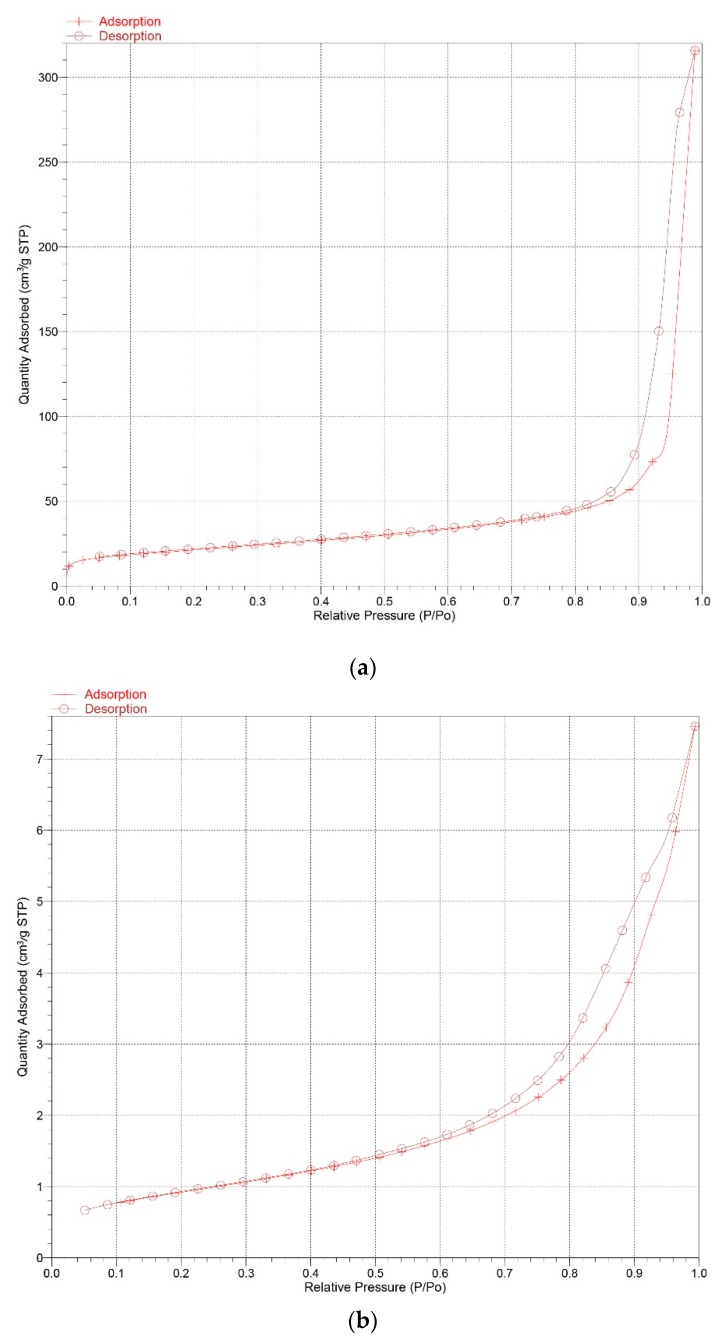
Isothermal adsorption curves of K-A-S-H samples (**a**) K-A-S-H 2.0_2_-7d-60, (**b**) K-A-S-H 2.0_2_-7d-95.

The particle size distribution of the K-A-S-H samples are shown in Figure 8. Additionally, the BET nitrogen adsorption method was used to determine the specific surface area of the synthesized K-A-S-H samples, the isothermal adsorption curve and specific surface area data are shown in Figure 9 and Table 2, separately.

The particle size distribution for each group of K-A-S-H samples was relatively stable, with a range of particle diameter of 100–300 nm, and an average particle size of about 180 nm. The results of particle size distribution analysis agree well with the results of sample agglomerated particle size obtained by TEM observation. The XRD, SEM, and TEM analysis of sample K-A-S-H2.0_2_-7d-60 revealed an amorphous product, with the narrowness of the particle size distribution range likely related to the increase in the homogeneity of the raw material that did not completely react.

Figure 9 shows the isotherm adsorption curve of samples K-A-S-H 2.0_2_-7d-60 (amorphous) and K-A-S-H 2.0_2_-7d-95 (crystalline). In these two curves, there exist the hysteresis loops, which is thought to be caused by the detaining of the N_2_ gas molecule in gel micropores in the desorption process. It is found that the area between crystalline K-A-S-H sample hysteresis loops decreases, indicating that the number of nitrogen molecules entering the crystalline K-A-S-H sample’s microstructural layer or gel micropores is less than that in the amorphous K-A-S-H sample. The calculated specific surface area data show a decrease in the BET specific surface area of the K-A-S-H samples resulting from an increase in the SiO_2_/Al_2_O_3_ or K_2_O/SiO_2_ ratio. With extended curing temperature and the curing time, the specific surface area was significantly reduced. Together with the above results from XRD, TG, and SEM analysis that indicate a shift in product morphology from amorphous to semi-crystalline to crystalline, the structure of the K-A-S-H products shrinks to become more compact.

## 4. Conclusions

This study stoichiometrically synthesized K-A-S-H gels using a hydrothermal method. To probe the state of Al in the inner structure, conditions including K_2_O/SiO_2_ ratio, SiO_2_/Al_2_O_3_ ratio, curing temperature and curing time were varied and their effects on the resulting gels were investigated, and the following conclusions can be drawn:

(1) XRD results demonstrated the presence of amorphous K-A-S-H gel and two crystalline products of zeolite K-H and K-G zeolite. The high K_2_O/SiO_2_ ratios led to the formation of K-G zeolite, while zeolite K-H in low ratios. For all samples cured at temperatures below 80 °C, the K-A-S-H products were completely amorphous. With increased curing temperature and time, the K-A-S-H products transformed from amorphous form to crystalline zeolite phase structure, with the reduced specific surface area. Combined with the TG results, the system contains more nonevaporated water or zeolite water for structural rearrangement, indicating a denser K-A-S-H product structure.

(2) The FTIR results show that an increase of the K_2_O/SiO_2_ ratio led to an increase in the amount of non-bridged oxygen atoms in the structure, which in turn caused a shift in the Si–O–T absorption peak to a lower wavenumber and a slight decrease in the tetrahedral polymerization degree. The ^27^Al and ^29^Si MAS-NMR spectra reveal that the Al exhibits mainly VI and IV coordination, and the silicate network shows a progressive polymerization degree with increasing curing temperature and time. Additionally, the absorption band of the asymmetric stretching vibration of T–O–Si (T = Si or Al) shifted to a lower wavenumber. The amorphous K-A-S-H gel contained Q_2_ and Q_2_ (1Al) silicate groups arranged in a linear long-chain structure, with a crystalline state that was mainly composed of a three-dimensional crosslinking unit Q_4_. Different K_2_O/SiO_2_ ratios result in different highly polymerized three-dimensional network-like zeolite structures in the crystalline K-A-S-H products.

(3) Amorphous K-A-S-H gels exhibited an irregular floc structure as observed under SEM. The crystallized product was interspersed with clusters of crystals and a large number of needle shape or filaments. The K-A-S-H gels observed under TEM showed random grain polymerization or a thin, wrinkled foil, which finally forms a short rod-like and flaky crystallized product. In addition, within a certain range, there was no significant effect of the SiO_2_/Al_2_O_3_ ratio on the structure of K-A-S-H products.

Low crystallinity K-A-S-H gels and zeolite crystalline powders were synthesized and the differences and similarities of their (micro) structures were analyzed. Our previous studies [3,7] have demonstrated the synthetic C-A-S-H gel can be regarded as a chemical intensification of the hardening effect in alkali-activated binders, which also refine the pore structures of the alkali-activated binders and provide a novel approach of alkali-activated binders shrinkage reduction at an early stage. Meanwhile, these gels and the hydration products of alkali-activated materials have similar composition and morphology. In view of this, the results found in this research further suggests this gel could be extended to low calcium systems, such as K(N)-A-S-H (K_2_O(Na_2_O)-Al_2_O_3_-SiO_2_-H_2_O), to be further applied to simulate and intensify the alkaline-activated binders. In this paper, it could be shown that the experimental design with a curing temperature of no more than 95 °C and a curing time of about 3 days is suitable for the synthesis of similar potential gels.

## Figures and Tables

**Table 1:** Mixing proportions used to prepare K_2_O-Al_2_O_3_-SiO_2_-H_2_O (K-A-S-H).

Sample	K_2_O/SiO_2_	SiO_2_/Al_2_O_3_	Water/Solid Ratio
K-A-S-H1.0_1_	1.0	1.0	5.0
K-A-S-H2.0_1_	2.0	1.0	5.0
K-A-S-H0.5_2_	0.5	2.0	5.0
K-A-S-H1.0_2_	1.0	2.0	5.0
K-A-S-H2.0_2_	2.0	2.0	5.0

**Table 2:** BET specific surface area of K-A-S-H samples (m^2^/g).

2.0_2_-7d-60	2.0_1_-3d-95	2.0_2_-3d-95	0.5_2_-7d-95	1.0_2_-7d-95	2.0_2_-7d-95
74.23	50.07	34.17	32.32	11.96	3.34
